# The Design of the Emission Layer for Electron Multipliers

**DOI:** 10.1186/s11671-021-03606-y

**Published:** 2021-10-07

**Authors:** Yuman Wang, Baojun Yan, Kaile Wen, Shulin Liu, Ming Qi, Binting Zhang, Jianyu Gu, Wenjing Yao

**Affiliations:** 1 School of Physics, Nanjing University, Nanjing, 210093 China; 2 State Key Laboratory of Particle Detection and Electronics, Institute of High Energy Physics, Chinese Academy of Sciences, Beijing, 100049 China; 3 University of Chinese Academy of Sciences, Beijing, 100049 China

**Keywords:** Electron multipliers, Secondary electron emission, Al_2_O_3_, MgO, ALD

## Abstract

The electron multipliers gain is closely related to the secondary electron emission coefficient (SEE) of the emission layer materials. The SEE is closely related to the thickness of the emission layer. If the emission layer is thin, the low SEE causes the low gain of electron multipliers. If the emission layer is thick, the conductive layer can't timely supplement charge to the emission layer, the electronic amplifier gain is low too. The electron multipliers usually choose Al_2_O_3_ and MgO film as the emission layer because of the high SEE level. MgO easy deliquescence into Mg(OH)_2_ Mg_2_(OH)_2_CO_3_ and MgCO_3_ resulting in the lower SEE level. The SEE level of Al_2_O_3_ is lower than MgO, but Al_2_O_3_ is stable. We designed a spherical system for testing the SEE level of materials, and proposed to use low-energy secondary electrons instead of low-energy electron beam for neutralization to measuring the SEE level of Al_2_O_3_, MgO, MgO/Al_2_O_3_, Al_2_O_3_/MgO, and precisely control the film thickness by using atomic layer deposition. We propose to compare the SEE under the adjacent incident electrons energy to partition the SEE value of the material, and obtain four empirical formulas for the relationship between SEE and thickness. Since the main materials that cause the decrease in SEE are Mg_2_(OH)_2_CO_3_ and MgCO_3_, we use the C element atomic concentration measured by XPS to study the deliquescent depth of the material. We propose to use the concept of transition layer for SEE interpretation of multilayer materials. Through experiments and calculations, we put forward a new emission layer for electron multipliers, including 2–3 nm Al_2_O_3_ buffer layer, 5–9 nm MgO main-body layer, 1 nm Al_2_O_3_ protective layer or 0.3 nm Al_2_O_3_ enhancement layer. We prepared this emission layer to microchannel plate (MCP), which significantly improved the gain of MCP. We can also apply this new emission layer to channel electron multiplier and separate electron multiplier.

## Introduction

The secondary electron emission coefficient (SEE) of a material is defined as the ratio of the emitted secondary electrons number to the incident electrons number on the material. The application field of secondary electrons is very wide, mainly divided into the field of electron multiplication, the field of material surface composition and structure analysis, and the field of suppressing micro-discharge. The field of electron multiplication includes channel electron multiplier (CEM), microchannel plate (MCP), separate electron multiplier, micro-pulse gun (MPG), dielectric window, atomic clocks, etc. [1–9]. The field of material surface composition and structure analysis includes transmission electron microscope (TEM), scanning electron microscope (SEM), auger electron spectrometer (AES), electron diffractometer, etc. [10–13]. The field of suppressing micro-discharge includes the electron cloud problem on the inner surface of the ring-accelerator, the reliability and life of high-power microwave vacuum devices in space, the dielectric window breakdown of high-power microwave sources, the charging/discharging problems on the surface of the spacecraft, etc. [1, 14].

Our main research area is the application field of electron multiplication. Electron multipliers consist of the substrate, the conductive layer and the emission layer. The incident electron hitting the emission layer leads to the generation of secondary electron from the emission layer. The secondary electron will be further accelerated by bias voltage to hit the emission layer and lead to more and more secondary electron, resulting in an electron avalanche and the emission of a cloud of electrons from the output. The emission layer lost a large amount of electric charge due to more and more secondary electron, so the conductive layer for the loss of the electron emission continuously provides the charge [15].

The SEE is closely related to the thickness of the emission layer. If the emission layer is thin, the low SEE causes the low gain of electron multipliers. If the emission layer is thick, the conductive layer can't timely supplement charge to the loss charge of the emission layer due to the electron avalanche, resulting in the low gain of the electron multipliers. The experiment experience that the emission layer between 5 and 15 nm is appropriate. Therefore, the gain of electron multipliers is closely related to the SEE level of the materials and the thickness of the emission layer. It becomes very important to study the thickness of the emission layer and the SEE level of the materials.

**Fig. 1:**
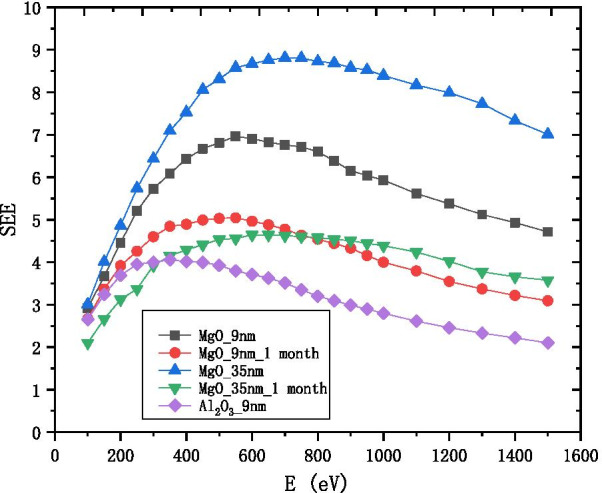
Variation of SEE of 9 nm-Al_2_O_3_ 9 nm-MgO and 35 nm-MgO with the incident electron energy, and the result measured after 1 month of air deliquescence of the sample

It is known that the SEE level of Al_2_O_3_ is very high [16]. Therefore, Al_2_O_3_ is usually selected as the emission layer film in the electron multipliers. But, the SEE level of MgO is much higher than Al_2_O_3_ [2, 17]. There are four reasons why MgO was not selected. First, MgO is easy to deliquesce into Mg(OH)_2_ Mg_2_(OH)_2_CO_3_ and MgCO_3_, which causes the SEE level to become as low as that of Al_2_O_3_ as shown in Fig. 1; second, the film will be very thick (35 nm) under the saturated SEE level of MgO, the conductive layer cannot replenish charge to the surface of the emission layer in time; third, the properties of Al_2_O_3_ are stable for a long time in the atmosphere; fourth, the preparation process of Al_2_O_3_ is simpler than that of MgO. Atomic layer deposition (ALD) can produce continuous no pin-microchannel film, have excellent coverage, and can control the atomic film thickness and composition. Therefore, we choose ALD as an important preparation method for studying the thickness of the emission layer [18–21].

It is known that the final products of deliquescent MgO are mainly Mg_2_(OH)_2_CO_3_ and MgCO_3_, so the content of C atom concentration at different depths of the material can reflect the deliquescent depth of MgO. The surface is etched by Ar ion beam sputtering and analyzed by X-ray photoelectron spectroscopy (XPS). The two are alternately performed. The etching depth is controlled by controlling the etching time, and the relative atomic concentration percentage changes of C and Mg elements are obtained by XPS. When XPS cannot measure the relative concentration percentage of C element, the etching depth at this time is the deliquescent depth of MgO. The above method shows that the deliquescent depth of MgO is about 3.8 nm and 1 nm Al_2_O_3_ can protect MgO from deliquescent.

In order to measure the SEE level of materials, many laboratories around the world have built their own dedicated measuring devices, including Stanford Linear Accelerator Center [14], the University of Utah [22], Princeton University [23]; ONERA/DESP [24]; University of Science and Technology of China, Xi’an Jiaotong University, 504 Institute of Aerospace, China Spallation Neutron Source, University Of Electronic Science And Technology Of China, etc. We designed a spherical system for testing the SEE level of materials to ensure the full collection of secondary electrons and help improve the accuracy of the measurement results. And, we recommend using low-energy secondary electrons instead of low-energy electron beams for neutralization to measure the SEE of insulating materials, such as MgO and Al_2_O_3_, it avoids the disadvantages of neutralization dose and neutralization time [24, 25], this method is convenient and low cost.

We designed the emission layer of the electron multiplier with the idea of building a house and achieved good results. We compare the SEE value under the incident electron energy of the neighbors, and use this as a standard to divide the material into a low-energy region, a medium-energy region and a high-energy region. This is different from the field of suppressing micro-discharge [14]. It is found that the middle energy region can eliminate the interference of incident electron energy on the SEE value. Therefore, the middle energy region is selected as the standard to measure the SEE level of the material, and Al_2_O_3_, MgO, MgO/Al_2_O_3_, Al_2_O_3_/MgO are studied to obtain the empirical formula.

The main SEE physical model currently proposed is the Dionne model [26, 27]. The proposed double-layer model [28] is further revised and is not suitable for the current experimental data. Therefore, we suggest using the concept of transition layer to explain multilayer materials, which can give a good explanation of the material characteristics of the design.

Our experiments and calculations found that after growing Al_2_O_3_ and then growing MgO, the saturated SEE level of MgO can be revealed when this film is thinner than the MgO film. This solves the problem that the MgO film is too thick and the conductive layer cannot supplement the charge for the emission layer. And we found that after growing MgO and then growing Al_2_O_3_, Al_2_O_3_ above 3 nm no longer shows the SEE level of MgO; the 1 nm Al_2_O_3_ can resist the damage of the external environment to MgO, and keep the SEE level of MgO for a long time; the 0.3 nm Al_2_O_3_ can raise the saturated SEE level of MgO. Therefore, we propose that the preparation process of the new emission layer is to grow a 9 nm MgO main layer on the 2 nm Al_2_O_3_ buffer layer, and then grow 1 nm Al_2_O_3_ protective layer or 0.3 nm Al_2_O_3_ enhancement layer on it, which can solve the problem of the MgO shortcomings of the emission layer in the electron multipliers. We have greatly improved the gain of the microchannel plate by growing this new type of emission layer in the microchannel of the microchannel plate (a kind of electron multiplier). The design thickness of this new emission layer is of great significance for improving the gain and stability of the electron multiplier.

## Experimental and Methods

### The Emission Layer Using Atomic Layer Deposition

Atomic layer deposition (ALD) is a kind of technology, which is the precursor gas and reaction gas alternately enter the basal surface at a controlled rate, physical or chemical adsorption on the surface or surface saturated reaction occurs on the surface, the material is deposited layer by layer in the form of a single atom film on the surface. ALD can produce continuous no pin-microchannel film, have excellent coverage, and can control the atomic film thickness and composition. Therefore, we choose ALD as an important preparation method for studying the thickness of the emission layer.

The following is the chemical reaction equation of using ALD to grow Al_2_O_3_:

$$\begin{aligned} {\text{A}} & :{\text{Substrate}} - {\text{OH}}^{*} + {\text{Al}}\left( {{\text{CH}}_{3} } \right)_{3} \to {\text{Substrate}} - {\text{O}} - {\text{Al}}\left( {{\text{CH}}_{3} } \right)_{2}^{*} + {\text{CH}}_{4} \uparrow \\ {\text{B}} & :{\text{Substrate}} - {\text{O}} - {\text{Al}}\left( {{\text{CH}}_{3} } \right)_{2}^{*} + 2{\text{H}}_{2} {\text{O}} \to {\text{Substrate}} - {\text{O}} - {\text{Al}}\left( {{\text{OH}}} \right)_{2}^{*} + 2{\text{CH}}_{4} \uparrow \\ {\text{C}} & :{\text{Al}} - {\text{OH}}^{*} + {\text{Al}}\left( {{\text{CH}}_{3} } \right)_{3} \to {\text{Al}} - {\text{O}} - {\text{Al}}\left( {{\text{CH}}_{3} } \right)_{2}^{*} + {\text{CH}}_{4} \uparrow \\ {\text{D}} & :{\text{Al}} - {\text{CH}}_{3}^{*} + {\text{H}}_{2} {\text{O}} \to {\text{Al}} - {\text{OH}}^{*} + 2{\text{CH}}_{4} \uparrow \\ \end{aligned}$$

**Fig. 2:**
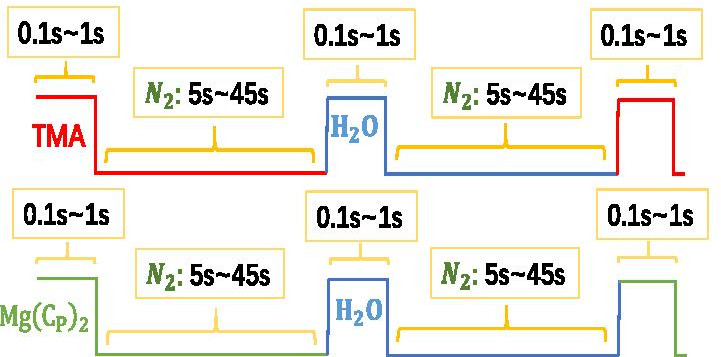
Schematic diagram of the growth process of Al_2_O_3_ and MgO

As the equation of A and B or C and D shown, the basal surface was originally covered with –OH, The chemical reaction of –OH and Al(CH_3_)_3_ (TMA) formed the new –CH_3_ surface, and released CH_4_ (byproduct). The new –CH_3_ surface exposed to water vapor, their reaction generated the new –OH surface and released CH_4_ again. The temperature of the reaction is 200 °C. The time and the order of growing a layer of Al_2_O_3_ atom as shown in Fig. 2:

$${\text{TMA/N}}_{2} {\text{/H}}_{2} {\text{O/N}}_{2} = 0.1\sim 1{\text{s}}/5\sim 45{\text{s}}/0.1\sim 1{\text{s }}/5\sim 45{\text{s}}{.}$$

The following is the chemical reaction equation of using ALD to grow MgO:

$$\begin{aligned} {\text{E}} & :{\text{Substrate}} - {\text{OH}}^{*} + {\text{Mg}}\left( {{\text{C}}_{5} {\text{H}}_{5} } \right)_{2} \to {\text{Substrate}} - {\text{O}} - {\text{MgC}}_{5} {\text{H}}_{5}^{*} + {\text{C}}_{5} {\text{H}}_{6} \uparrow \\ {\text{F}} & :{\text{Substrate}} - {\text{O}} - {\text{MgC}}_{5} {\text{H}}_{5}^{*} + {\text{H}}_{2} {\text{O}} \to {\text{Substrate}} - {\text{OH}}^{*} + {\text{C}}_{5} {\text{H}}_{6} \uparrow \\ {\text{G}} & :{\text{Mg}} - {\text{OH}}^{*} + {\text{Mg}}\left( {{\text{C}}_{5} {\text{H}}_{5} } \right)_{2} \to {\text{Mg}} - {\text{O}} - {\text{MgC}}_{5} {\text{H}}_{5}^{*} + {\text{C}}_{5} {\text{H}}_{6} \uparrow \\ {\text{H}} & :{\text{Mg}} - {\text{C}}_{5} {\text{H}}_{5}^{*} + {\text{H}}_{2} {\text{O}} \to {\text{Mg}} - {\text{OH}}^{*} + {\text{C}}_{5} {\text{H}}_{6} \uparrow \\ \end{aligned}$$

As the equation of E and F or G and H shown, the basal surface was originally covered with $- {\text{OH}}$, The chemical reaction of $- {\text{OH}}$ and ${\text{Mg}}\left( {{\text{C}}_{5} {\text{H}}_{5} } \right)_{2}$(${\text{Mg}}\left( {{\text{C}}_{{\text{P}}} } \right)_{2}$) formed the new $- {\text{C}}_{5} {\text{H}}_{5}$ surface, and released ${\text{C}}_{5} {\text{H}}_{6}$ (byproduct). The new $- {\text{C}}_{5} {\text{H}}_{5}$ surface exposed to water vapor, their reaction generated the new $- {\text{OH}}$ surface and released ${\text{C}}_{5} {\text{H}}_{6}$ again.

We heat ${\text{Mg}}\left( {{\text{C}}_{{\text{P}}} } \right)_{2}$ at 60 °C to turn it into dust. Temperature of the reaction chamber is 200 °C. The time and order of growing a layer of MgO atom as shown in Fig. 2:

$${\text{Mg}}\left( {{\text{Cp}}} \right)_{2} {\text{/N}}_{2} {\text{/H}}_{2} {\text{O/N}}_{2} = 0.1\sim 1{\text{s}}/5\sim 45{\text{s}}/0.1\sim 1{\text{s }}/5\sim 45{\text{s}}{.}$$

### The Design of Emission Layer

**Fig.3:**
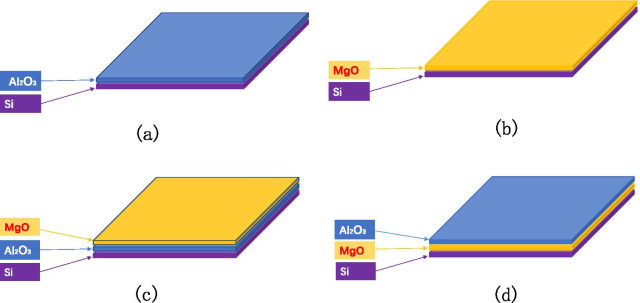
Research on the relationship between film thickness and SEE by designing the emission layer experiment

The samples are prepared in four ways as shown in Fig. 3: grow different thicknesses of ${\text{Al}}_{2} {\text{O}}_{3}$ on Si wafer; grow different thicknesses of MgO on Si wafer; grow different thicknesses of ${\text{Al}}_{2} {\text{O}}_{3}$ on Si wafer and then grow fixed thicknesses of MgO; grow a fixed thickness of MgO on the Si wafer and then grow a different thickness of ${\text{Al}}_{2} {\text{O}}_{3}$. We have grown different thicknesses of ${\text{Al}}_{2} {\text{O}}_{3}$ on Si wafer (1 nm, 3 nm, 7 nm, 9 nm, 30 nm, 50 nm). We have grown different thicknesses of MgO on Si wafer (1 nm, 3 nm, 5 nm, 9 nm, 15 nm, 20 nm, 35 nm). We grow different thicknesses of ${\text{Al}}_{2} {\text{O}}_{3}$ on Si wafer (0.6 nm, 1 nm, 3 nm, 30 nm) and then grow fixed thicknesses of MgO (9 nm).We grow a fixed thickness of MgO on the Si wafer (35 nm) and then grow a different thickness of ${\text{Al}}_{2} {\text{O}}_{3}$ (0.3 nm, 0.6 nm, 1 nm, 3 nm, 5 nm, 7 nm, 10 nm, 20 nm).

### The New Test Method for SEE

**Fig.4:**
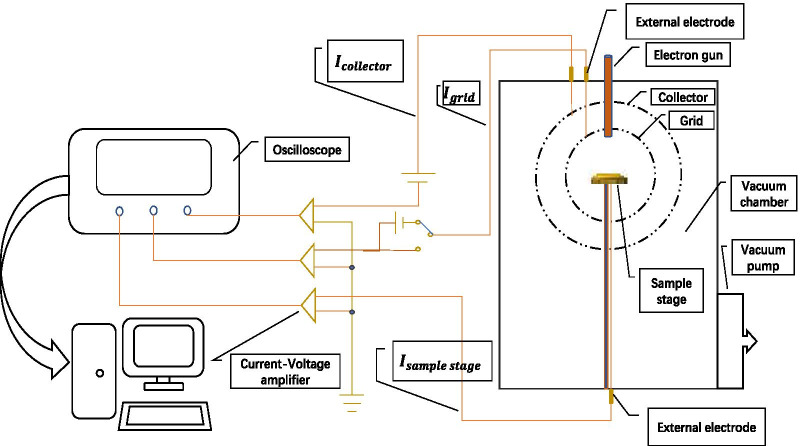
Schematic diagram of secondary electron emission efficiency system

We use the collector method to measure as shown in Fig. 4: first connect the sample stage to the collector, the current measured by the picoammeter is the incident electron current, denoted as $I_{{\text{p}}}$; under the same incident conditions, disconnect the sample and collector, at this time the measured current on the collector is the secondary electron current, denoted as $I_{{\text{s}}}$.

$${\text{SEE}} = \frac{{I_{{\text{s}}} }}{{I_{{\text{p}}} }}$$

We designed the device into a global-shaped structure to ensure the full collection of secondary electrons and help improve the accuracy of the measurement results.

When the insulating material is bombarded by incident electrons, the surface of the material emits secondary electrons and accumulates positive charges due to the loss of electrons. The positive charge makes the potential rise. Because the secondary electrons are generated within a few nanometers of the material surface and have low energy (~ eV). The secondary electrons are very susceptible to the positive potential. The positive potential will affect the next secondary electron emission process leading to a decline in secondary electron yield.

In order to eliminate the effect of charge accumulation on the measurement result of the SEE of the insulating sample and accurately measure the SEE of the insulating sample, the traditional method directly uses a low-energy electron beam to irradiate the insulating sample, and the positive charge on the surface of the sample is neutralized by the low-energy electron. The traditional method has two disadvantages. First, it needs to accurately calculate the neutralization dose, it is easy to have positive charge on the sample surface due to insufficient neutralization dose, or negative charge on the sample surface due to excessive neutralization; second, it needs to be equipped with another one low energy electron gun [24, 25].

**Fig.5:**
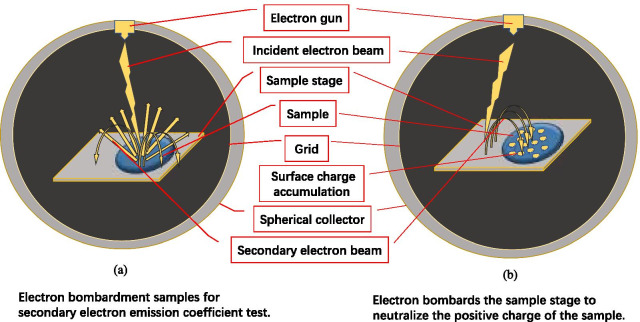
Schematic diagram of the new test method for the secondary electron emission coefficient of the material

We propose to use low-energy secondary electrons instead of low-energy electron beam for neutralization, which overcomes the shortcomings of traditional methods and obtains accurate secondary electrons as shown in Fig. 5 [29]. We place the insulation sample to be tested on half of the sample stage, and leave the other half empty. The sample table is made of 304 stainless steel, and the electric potential is 0 V.

When testing an insulating sample, the electrons generated by the electron gun bombard the surface of the insulating sample as shown in Fig. 5a, resulting in a positive charge area as shown in Fig. 5b. When neutralizing the surface charge of the insulating sample, the half empty area of the sample stage is bombarded by adjusting the angle of the electron gun to make the sample stage emit secondary electrons as shown in Fig. 5b.

Due to the mutual attraction of positive charges and electrons, secondary electrons are attracted to the sample surface for charge neutralization. As the positive charge decreases, fewer electrons are attracted. When the positive charge on the sample surface is neutralized, the surface of the insulating sample returns to its original state. Because there is no positive charge, it will not continue to attract the low-energy secondary electrons generated by the sample stage, so there will be no excessive neutralization that causes the sample surface to be negatively charged.

**Fig.6:**
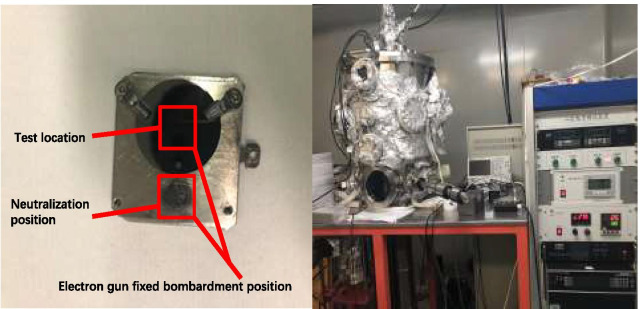
Photographs of the sample, the sample stage, and the secondary electron emission coefficient test equipment

The electron gun we use bombards the sample surface at the same position each time, and then deflects the same angle to bombard the same position on the sample stage as shown in Fig. 6. Due to the long-term SEE test process, the position on the sample stage bombarded by the electron gun for a long time became a black spot as shown in Fig. 6.

## Result and Discussion

### SEE Zoning and Analysis

We compare the SEE value under the adjacent incident electrons energy to describe the change of SEE with the energy of incident electrons and define it as

$$R_{{{\text{SEE}}}} = \frac{{{\text{SEE}}\left( {x + b } \right)}}{{{\text{SEE}}\left( {x } \right)}}$$

and the SEE of the material is divided into three areas by the size of the $R_{{{\text{SEE}}}}$ value, namely the low energy region of the incident electron ($R_{{{\text{SEE}}}} \ge 1.02$), the medium energy region of the incident electron ($0.98 \le R_{{{\text{SEE}}}} < 1.02$) and the high energy region of the incident electron (${\text{R}}_{{{\text{SEE}}}} \ge 0.98$). The incident electron energy range of the material we use to test SEE is (100 eV, 1500 eV), *x* represents the incident electron energy, and *b* represents the step length of the incident electron energy in the SEE test.

**Fig.7:**
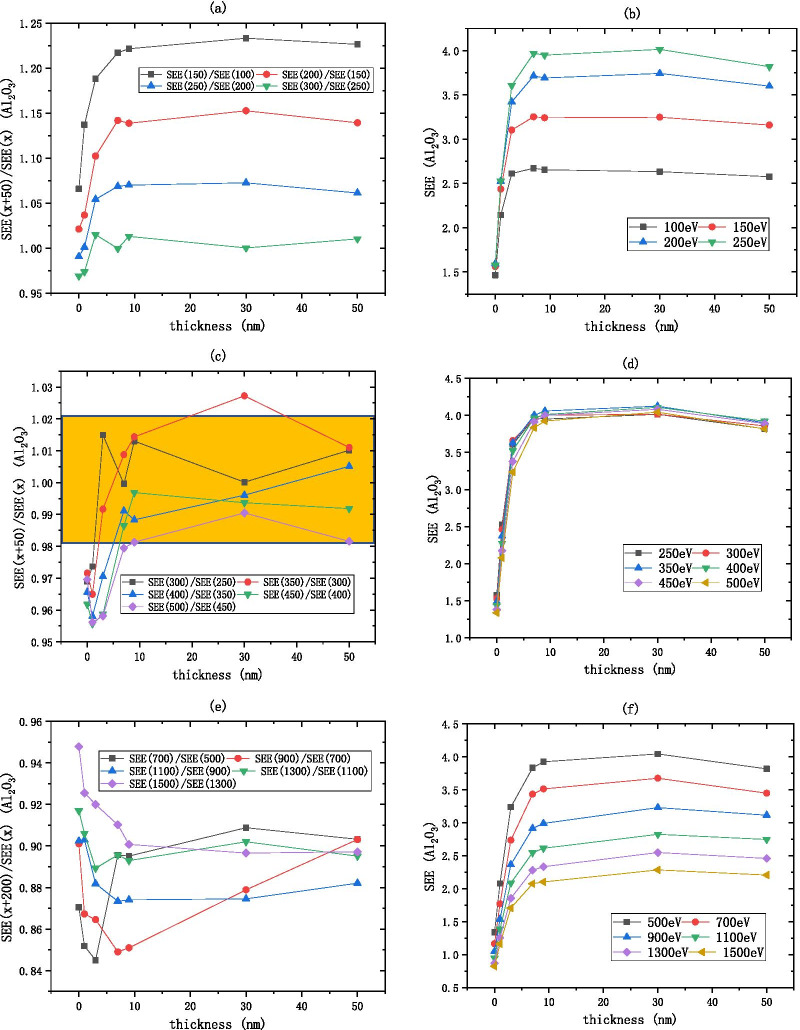
After dividing the incident electron energy by $R_{{{\text{SEE}}}} = \frac{{\text{SEE(x + b)}}}{{\text{SEE(x)}}}$ as shown in the **a**, **c**, **e** the change of Al_2_O_3_ (on the silicon wafer, grow xnm-Al_2_O_3_) SEE with thickness as shown in the **b**, **d**, **f**

${\text{Al}}_{2} {\text{O}}_{3}$ SEE basically remains unchanged after 7 nm as shown in Fig. 7. As shown in Fig. 7a, b, the low energy region of ${\text{Al}}_{2} {\text{O}}_{3}$ is between 100 and 250 eV, the $R_{{{\text{SEE}}}}$ decreases from 1.25 to 1.02, indicating that as the incident electron energy increases, the SEE increases and finally stabilizes. As shown in Fig. 7c, d, the medium energy region of ${\text{Al}}_{2} {\text{O}}_{3}$ is between 250 and 500 eV, the $R_{{{\text{SEE}}}}$ is considered constant within the interval of [0.98, 1.02], that is, the $R_{{{\text{SEE}}}}$ is approximately equal to 1, indicating that the SEE is basically unchanged as the incident electron energy increases. As shown in Fig. 7e, f, the high energy region of ${\text{Al}}_{2} {\text{O}}_{3}$ is between 500 and 1500 eV, for every increase of 200 eV of incident electron energy, the SEE decreases by about 0.9 times.

**Fig. 8:**
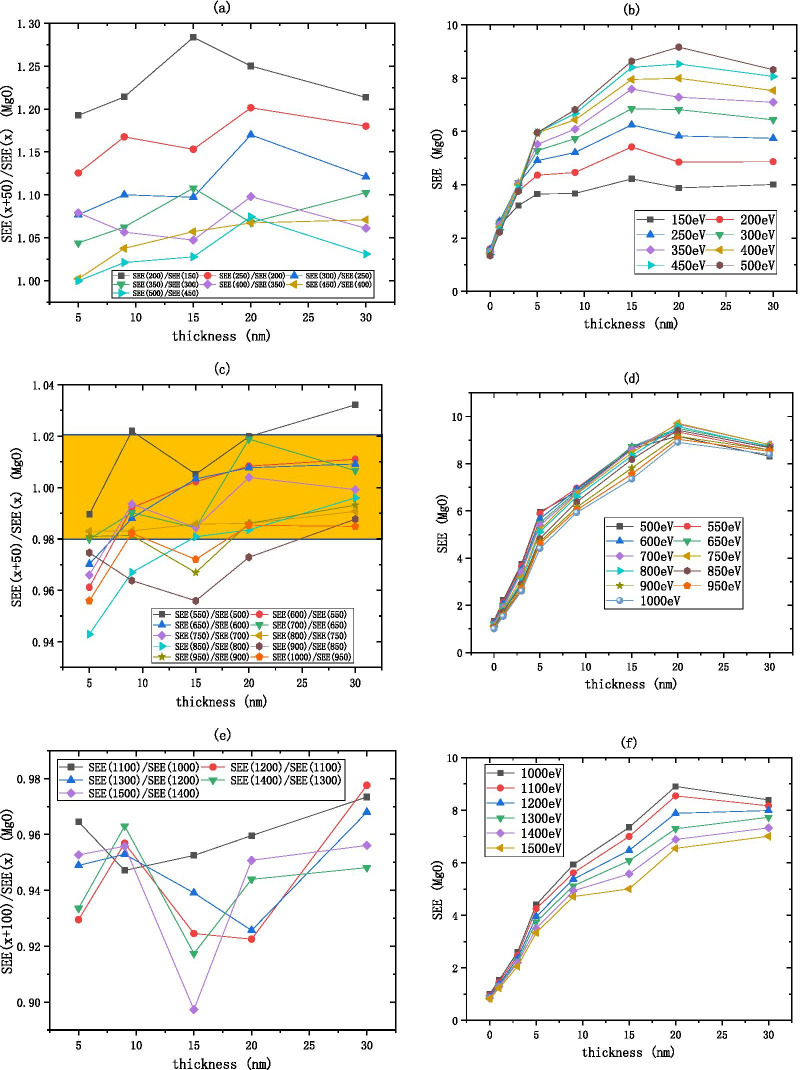
After dividing the incident electron energy by $R_{{{\text{SEE}}}} = \frac{{\text{SEE(x + b)}}}{{\text{SEE(x)}}}$ as shown in the **a**, **c**, **e** the change of MgO (on the silicon wafer, grow xnm-MgO) SEE with thickness as shown in the **b**, **d**, **f**

**Fig. 9:**
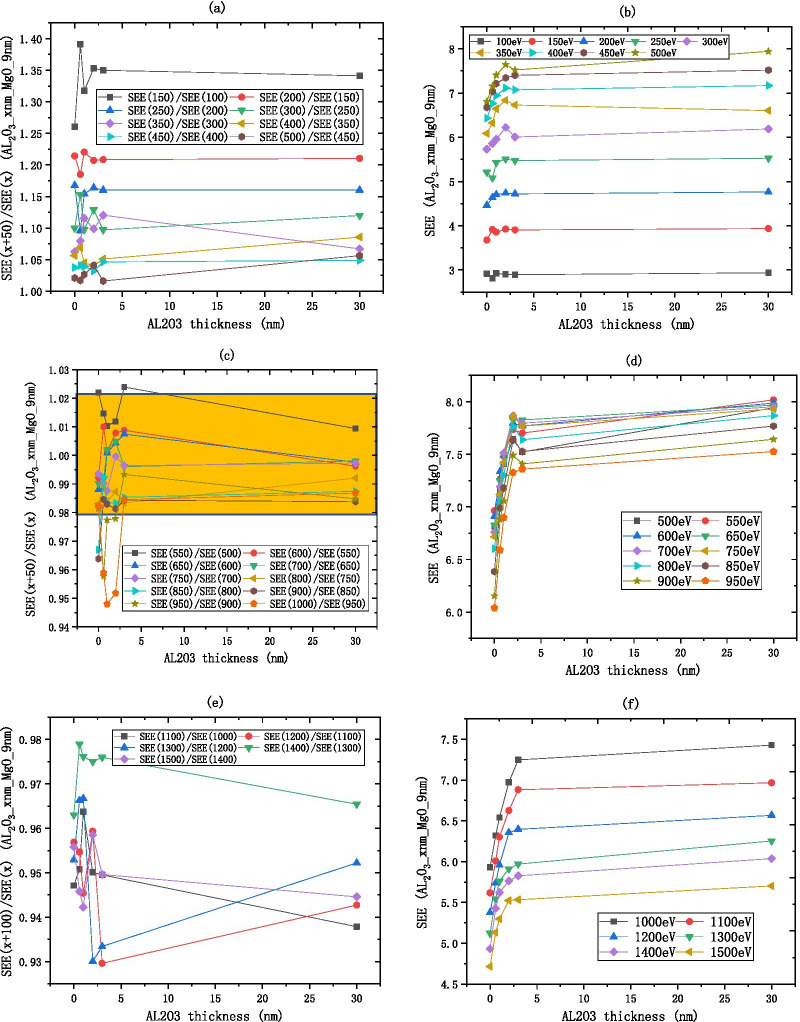
After dividing the incident electron energy by $R_{{{\text{SEE}}}} = \frac{{\text{SEE(x + b)}}}{{\text{SEE(x)}}}$ as shown in the **a**, **c**, **e** the change of Al_2_O_3_/MgO (on the silicon wafer, grow xnm-Al_2_O_3_ and then grow 9 nm-MgO) SEE with thickness as shown in the **b**, **d**, **f**

The MgO SEE basically remains unchanged after 20 nm as shown in Fig. 9. As shown in Fig. 8a, b, the low energy region of MgO is between 100 and 500 eV, the $R_{{{\text{SEE}}}}$ decreases from 1.3 to 1, indicating that as the incident electron energy increases, the SEE increases and finally stabilizes. As shown in Fig. 8c, d, the medium energy region of MgO is between 500 and 1000 eV, the $R_{{{\text{SEE}}}}$ is considered constant within the interval of [0.98, 1.02], that is, the $R_{{{\text{SEE}}}}$ is approximately equal to 1, indicating that the SEE is basically unchanged as the incident electron energy increases. As shown in Fig. 8e, f, the high energy region of MgO is between 1000 and 1500 eV, for every increase of 100 eV of incident electron energy, the SEE decreases by about 0.94 times.

As shown in Fig. 9, The SEE of ${\text{Al}}_{2} {\text{O}}_{3}$/MgO and MgO have similar incident electron energy partition, the SEE of ${\text{Al}}_{2} {\text{O}}_{3}$/MgO basically remains unchanged after 3 nm. As shown in Fig. 9a, b, the low energy region of ${\text{Al}}_{2} {\text{O}}_{3}$/MgO is between 100 and 450 eV, the $R_{{{\text{SEE}}}}$ decreases from 1.4 to 1.05, indicating that as the incident electron energy increases, the SEE increases and finally stabilizes. As shown in Fig. 9c, d, the medium energy region of ${\text{Al}}_{2} {\text{O}}_{3}$/MgO is between 500 and 1000 eV, the $R_{{{\text{SEE}}}}$ is considered constant within the interval of [0.98, 1.02], that is, the $R_{{{\text{SEE}}}}$ is approximately equal to 1, indicating that the SEE is basically unchanged as the incident electron energy increases. As shown in Fig. 9e, f, the high energy region of ${\text{Al}}_{2} {\text{O}}_{3}$/MgO is between 1000 and 1500 eV, for every increase of 100 eV of incident electron energy, the SEE decreases by about 0.95 times. Because the SEE of ${\text{Al}}_{2} {\text{O}}_{3}$/MgO is stable in the medium energy region, the incident electron energy can be excluded as a variable factor.

**Fig.10:**
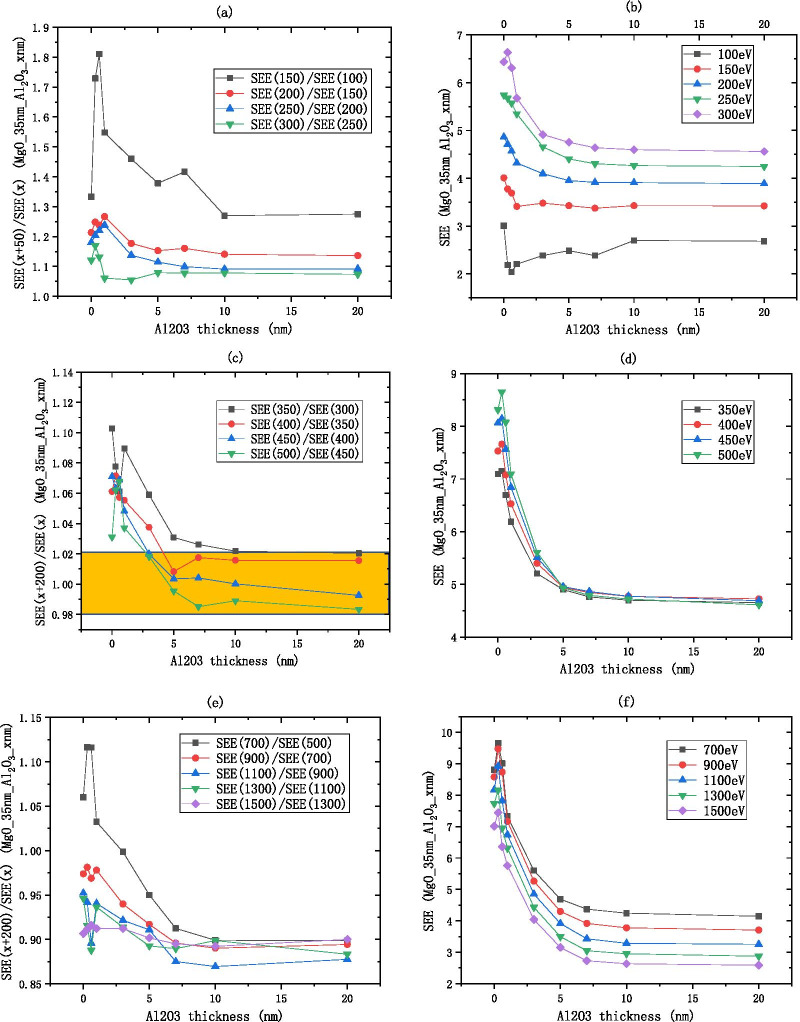
After dividing the incident electron energy by $R_{{{\text{SEE}}}} = \frac{{\text{SEE(x + b)}}}{{\text{SEE(x)}}}$ as shown in the **a, c, e**, the change of MgO/Al_2_O_3_ (on the silicon wafer, grow 35 nm-MgO, and then grow xnm-Al_2_O_3_) SEE with thickness as shown in the **b**, **d, f**

As shown in Fig. 10, The SEE of MgO/${\text{Al}}_{2} {\text{O}}_{3}$ and ${\text{Al}}_{2} {\text{O}}_{3}$ have similar incident electron energy partition, the SEE of MgO/${\text{Al}}_{2} {\text{O}}_{3}$ basically remains unchanged after 3 nm. As shown in Fig. 10a, b, the low energy region of MgO/${\text{Al}}_{2} {\text{O}}_{3}$ is between 100 and 300 eV, the $R_{{{\text{SEE}}}}$ decreases from 1.8 to 1, indicating that as the incident electron energy increases, the SEE increases and finally stabilizes. As shown in Fig. 10c, d, the medium energy region of MgO/${\text{Al}}_{2} {\text{O}}_{3}$ is between 300 and 500 eV, the $R_{{{\text{SEE}}}}$ is considered constant within the interval of [0.98, 1.02], when ${\text{Al}}_{2} {\text{O}}_{3}$ is thin, $R_{{{\text{SEE}}}}$ deviates from 1, and the difference in SEE under different incident electron energies is obvious; when ${\text{Al}}_{2} {\text{O}}_{3}$ is thick, $R_{{{\text{SEE}}}}$ is close to 1, and the difference is not obvious. As shown in Fig. 10e, f, the high energy region of MgO/${\text{Al}}_{2} {\text{O}}_{3}$ is between 500 and 1500 eV, when ${\text{Al}}_{2} {\text{O}}_{3}$ is thin, $R_{{{\text{SEE}}}}$ is close to 1, and the difference in SEE under different incident electron energies is not obvious; when ${\text{Al}}_{2} {\text{O}}_{3}$ is thick, $R_{{{\text{SEE}}}}$ deviates from 1, and the difference is obvious; for every increase of 200 eV of incident electron energy, the SEE decreases by about 0.9 times.

**Fig.11:**
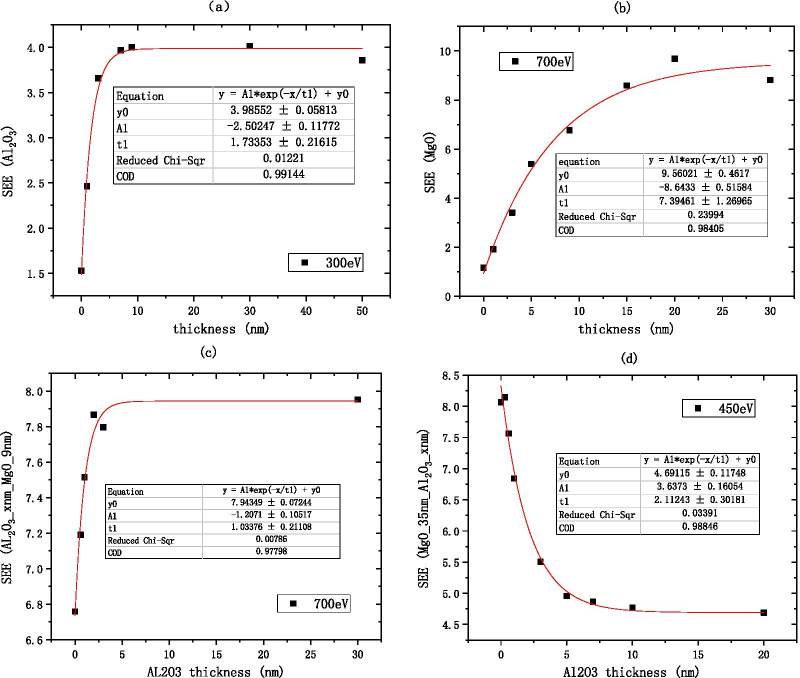
Relationship between the material's best secondary electron emission coefficient and film thickness, **a** shows the information of Al_2_O_3_ (on the silicon wafer, grow xnm-Al_2_O_3_), **b** shows the information of MgO (on the silicon wafer, grow xnm-MgO), **c** shows the information of Al_2_O_3_/MgO (on the silicon wafer, grow xnm-Al_2_O_3_, and then grow 9 nm-MgO), and **d** shows the information of MgO/Al_2_O_3_ (on the silicon wafer, grow 35 nm-MgO, and then grow xnm-Al_2_O_3_)

Because the ${\text{Al}}_{2} {\text{O}}_{3}$ SEE is stable in the medium energy region, the incident electron energy can be excluded as a variable factor. We choose the medium incident electron energy 300 eV as the standard to measure the SEE level of ${\text{Al}}_{2} {\text{O}}_{3}$, the empirical formula for the thickness of ${\text{Al}}_{2} {\text{O}}_{3}$ and the best SEE is obtained by fitting as shown in Fig. 11a (Table 1).

1 $${\text{B}}\_{\text{SEE}}_{{{\text{Al}}_{2} {\text{O}}_{3} }} = 3.99 - 2.5{*}e^{{ - \frac{{{\text{thickness}}}}{1.73}}}$$

**Table 1:** Incident electron energy partition of different materials, and the empirical formula for the best SEE and thickness of the material

Incident electron energy partition	Al_2_O_3_ (eV)	MgO (eV)	Al_2_O_3_/MgO (eV)	MgO/Al_2_O_3_ (eV)
Low	[0, 250]	[0, 500]	[0, 500]	[0, 300]
Medium	[250, 500]	[500, 1000]	[500, 1000]	[500, 1000]
High	[500, 1500]	[1000, 1500]	[1000, 1500]	[500, 1500]
Best SEE formula	3.99 − 205 * *e*^thickness/1.73^	9.56 – 8.64 * *e*^thickness/7.39^	7.94 – 1.21 * *e*^thickness/1.03^	4.69 + 3.64 * *e*^thickness/2.11^

Because the MgO SEE is stable in the medium energy region, the incident electron energy can be excluded as a variable factor. We choose the medium incident electron energy 700 eV as the standard to measure the SEE level of MgO, the empirical formula for the thickness of alumina material and the best SEE is obtained by fitting as shown in Fig. 11b.

2 $${\text{B}}\_{\text{SEE}}_{{{\text{MgO}}}} = 9.56 - 8.64*e^{{ - \frac{{{\text{thickness}}}}{7.39}}}$$

Because the SEE of ${\text{Al}}_{2} {\text{O}}_{3} /{\text{MgO}}$ is stable in the medium energy region, the incident electron energy can be excluded as a variable factor. We choose the medium incident electron energy 700 eV as the standard to measure the SEE level of ${\text{Al}}_{2} {\text{O}}_{3}$/MgO, the empirical formula for the thickness of alumina material and the best SEE is obtained by fitting as shown in Fig. 11c.

3 $${\text{B}}\_{\text{SEE}}_{{{\text{Al}}_{2} {\text{O}}_{3} /{\text{MgO}}}} = 7.94 - 1.21\,*\,e^{{ - \frac{{{\text{thickness}}}}{1.03}}}$$

Because the SEE of MgO/${\text{Al}}_{2} {\text{O}}_{3}$ is stable in the medium energy region, the incident electron energy can be excluded as a variable factor. We choose the medium incident electron energy 450 eV as the standard to measure the SEE level of MgO/${\text{Al}}_{2} {\text{O}}_{3}$, the empirical formula for the thickness of alumina material and the best SEE is obtained by fitting as shown in Fig. 11d.

4 $${\text{B}}\_{\text{SEE}}_{{{\text{MgO}}/{\text{Al}}_{2} {\text{O}}_{3} }} = 4.69 + 3.64\,*\,e^{{ - \frac{{{\text{thickness}}}}{2.11}}}$$

$$\frac{{{\text{B}}\_{\text{SEE}}_{{{\text{MgO}}}} \left( 9 \right)}}{{{\text{B}}\_{\text{SEE}}_{{{\text{Al}}_{2} {\text{O}}_{3} }} \left( {30} \right)}} = \frac{{9.56 - 8.64\,*\,e^{{ - \frac{9}{7.39}}} }}{{3.99 - 2.5\,*\,e^{{ - \frac{30}{{1.73}}}} }} \approx 1.755$$

According to formulas 1 and 2, the SEE level of 9 nm MgO is 1.755 times higher than that of 30 nm ${\text{Al}}_{2} {\text{O}}_{3}$.

$$\begin{aligned} \frac{{{\text{B}}\_{\text{SEE}}_{{{\text{Al}}_{2} {\text{O}}_{3} /{\text{MgO}}}} \left( 3 \right)}}{{{\text{B}}\_{\text{SEE}}_{{{\text{Al}}_{2} {\text{O}}_{3} }} \left( {30} \right)}} & = \frac{{7.94 - 1.21\,*\,e^{{ - \frac{3}{{1.03}}}} }}{{3.99 - 2.5\,*\,e^{{ - \frac{{30}}{{1.73}}}} }} \approx 1.973 \\ \frac{{{\text{B}}\_{\text{SEE}}_{{{\text{Al}}_{2} {\text{O}}_{3} /{\text{MgO}}}} \left( 3 \right)}}{{{\text{B}}_{{{\text{SEE}}\,{\text{MgO}}}} \left( 9 \right)}} & = \frac{{7.94 - 1.21\,*\,e^{{ - \frac{3}{{1.03}}}} }}{{9.56 - 8.64\,*\,e^{{ - \frac{9}{{7.39}}}} }} \approx 1.124 \\ \end{aligned}$$

**Fig.12:**
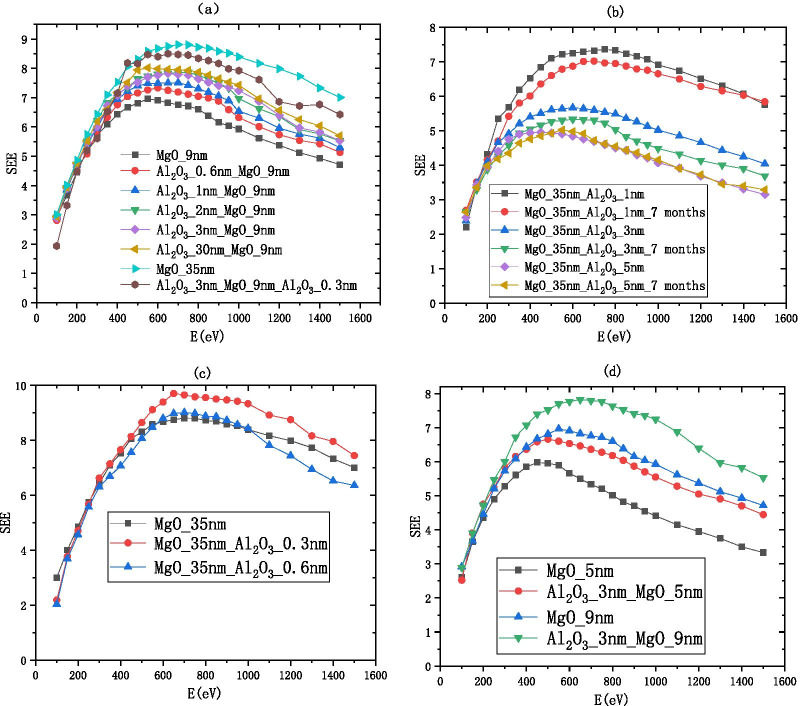
Change of secondary electron emission coefficient with different incident electron energy, **a** shows the information of Al_2_O_3_/MgO (on the silicon wafer, grow xnm-Al_2_O_3_, and then grow 9 nm-MgO), **b** shows the information of MgO/Al_2_O_3_ and deliquescent MgO/Al_2_O_3_ (on the silicon wafer, grow 35 nm-MgO, and then grow 1 nm-Al_2_O_3_), **c** shows the information of MgO/Al_2_O_3_ (on the silicon wafer, grow 35 nm-MgO, and then grow 0.3 nm-Al_2_O_3_), and **d** shows the information of Al_2_O_3_/MgO (on the silicon wafer, grow 3 nm-Al_2_O_3_, and then grow 5 nm-MgO)

We deposit 0–30 nm ${\text{Al}}_{2} {\text{O}}_{3}$ and redeposit 9 nm MgO on the Si wafer as the film, as shown in Fig. 12a. formulas 1 and 3 show that the SEE level of 9 nm MgO grown on 3 nm ${\text{Al}}_{2} {\text{O}}_{3}$ is 1.973 times higher than that of ${\text{Al}}_{2} {\text{O}}_{3}$. formulas 2 and 3 show that the SEE level of 9 nm MgO grown on 3 nm ${\text{Al}}_{2} {\text{O}}_{3}$ is 1.124 times higher than that of 9 nm MgO.

$$\frac{{{\text{B}}\_{\text{SEE}}_{{{\text{MgO}}/{\text{Al}}_{2} {\text{O}}_{3} }} }}{{{\text{B}}\_{\text{SEE}}_{{{\text{Al}}_{2} {\text{O}}_{3} }} }} = \frac{{4.69 + 3.64\,*\,e^{{ - \frac{1}{2.11}}} }}{{3.99 - 2.5\,*\,e^{{ - \frac{30}{{1.73}}}} }} \approx 1.743$$

The SEE level of MgO after deliquescent drops significantly as shown in Fig. 1. Then, we deposit 35 nm MgO and redeposit 1 nm ${\text{Al}}_{2} {\text{O}}_{3}$ on the Si wafer as the film. We found the SEE of this film exposed to the air 7 months is close to the SEE without exposed to the air as shown in Fig. 12b. Formulas 1 and 3 show that the SEE level of 1 nm ${\text{Al}}_{2} {\text{O}}_{3}$ grown on MgO is 1.743 times higher than the SEE of ${\text{Al}}_{2} {\text{O}}_{3}$ and can be long-term maintain a high SEE level (no obvious deliquescence in 7 months).

$$\begin{aligned} \frac{{{\text{B}}\_{\text{SEE}}_{{{\text{MgO}}/{\text{Al}}_{2} {\text{O}}_{3} }} \left( {0.3} \right)}}{{{\text{B}}\_{\text{SEE}}_{{{\text{Al}}_{2} {\text{O}}_{3} }} \left( {30} \right)}} & = \frac{{4.69 + 3.64\,*\,e^{{ - \frac{0.3}{{2.11}}}} }}{{3.99 - 2.5\,*\,e^{{ - \frac{30}{{1.73}}}} }} \approx 1.967, \\ \frac{{{\text{B}}\_{\text{SEE}}_{{{\text{MgO}}/{\text{Al}}_{2} {\text{O}}_{3} }} \left( {0.3} \right)}}{{{\text{B}}\_{\text{SEE}}_{{{\text{MgO}}}} \left( 9 \right)}} & = \frac{{4.69 + 3.64\,*\,e^{{ - \frac{0.3}{{2.11}}}} }}{{9.56 - 8.64\,*\,e^{{ - \frac{9}{7.39}}} }} \approx 1.12 \\ \end{aligned}$$

We deposited 35 nm MgO on the Si wafer and re-deposited 0.3 nm ${\text{Al}}_{2} {\text{O}}_{3}$ as a thin film as shown in Fig. 12c. It can be seen from formulas 1, 2 and 4 that the SEE level of 0.3 nm ${\text{Al}}_{2} {\text{O}}_{3}$ grown on MgO is 1.967 times higher than that of ${\text{Al}}_{2} {\text{O}}_{3}$ and 1.12 times higher than that of MgO;

The emission layer of the electron multiplier pursues thinner and higher SEE level, so we sacrificed some SEE level to make the film thinner. We deposited 3 nm ${\text{Al}}_{2} {\text{O}}_{3}$ on the Si wafer and re-deposited 5 nm MgO as a thin film as shown in Fig. 12d.

**Fig.13:**
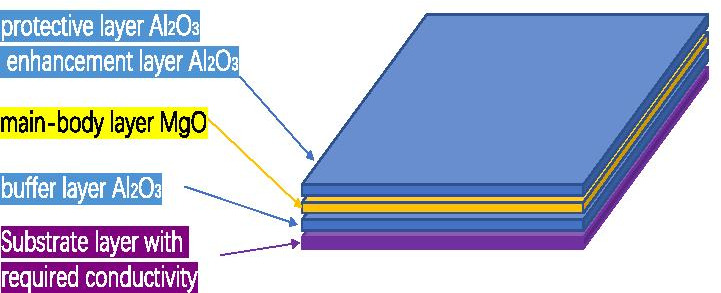
Schematic diagram of sandwich structure (Al_2_O_3_/MgO/Al_2_O_3_)

**Fig.14:**
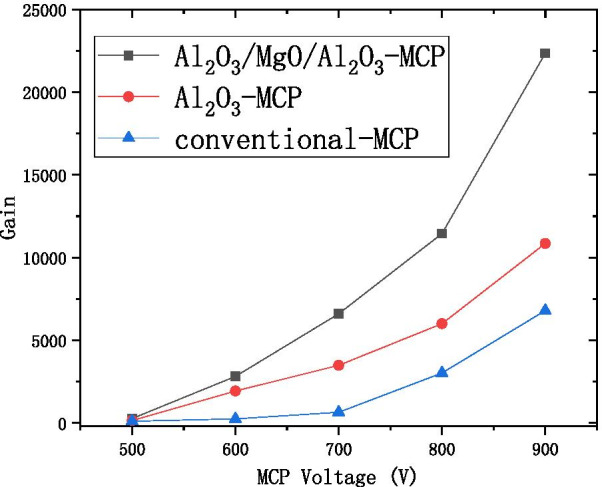
Relationship between the voltage and gain of the three microchannel plates (conventional microchannel plate, microchannel plate for growing Al_2_O_3_ emission layer, microchannel plate for growing Al_2_O_3_/MgO/Al_2_O_3_ emission layer)

We propose to grow 2–3 nm ${\text{Al}}_{2} {\text{O}}_{3}$ as a buffer layer, grow 5–9 nm MgO as the main layer, and grow 0.3 nm ${\text{Al}}_{2} {\text{O}}_{3}$ as an enhancement layer or 1 nm ${\text{Al}}_{2} {\text{O}}_{3}$ as a protective layer as the ${\text{Al}}_{2} {\text{O}}_{3}$/MgO/${\text{Al}}_{2} {\text{O}}_{3}$ emissive layer of electron multipliers as shown in Fig. 13. SEE level of ${\text{Al}}_{2} {\text{O}}_{3}$/MgO/${\text{Al}}_{2} {\text{O}}_{3}$ emission layer (${\text{Al}}_{2} {\text{O}}_{3}$/MgO/${\text{Al}}_{2} {\text{O}}_{3}$ = 3 nm/9 nm/0.3 nm) is shown in Fig. 12a. And, we tested a traditional microchannel plate with good gain and then grew ${\text{Al}}_{2} {\text{O}}_{3}$/MgO/${\text{Al}}_{2} {\text{O}}_{3}$ emission layer on microchannel wall of microchannel plate, and the gain result obtained by the test was significantly improved. Then, another piece of the first convention microchannel plate with close gain is grown with ${\text{Al}}_{2} {\text{O}}_{3}$ emission layer. Compared with the gain results obtained by the test, the ${\text{Al}}_{2} {\text{O}}_{3}$/MgO/${\text{Al}}_{2} {\text{O}}_{3}$ emission layer structure is more superior as shown in Fig. 14.

### XPS Characterization and Transition Layer Concept

SEE data usually uses Dionne model for fitting analysis [26, 27]. The current double-layer model based on Dionne model does not consider the existence of a transition layer between the two materials. Through the design of the emission layer structure this time, the SEE difference between ${\text{Al}}_{2} {\text{O}}_{3}$/MgO and ${\text{Si}}$/MgO can be clearly observed. Under the same SEE level, MgO exhibits a very large thickness difference. Sample (0.3 nm ${\text{Al}}_{2} {\text{O}}_{3}$ grown on MgO) can get a higher SEE than MgO. Sample (1 nm ${\text{Al}}_{2} {\text{O}}_{3}$ grown on MgO) maintain a high SEE level. The current double-layer model [28] can no longer explain the above phenomenon, so we put forward the concept of transition layer, there are two kinds of materials at the interface, forming two processes: the process of destroying the bottom material and the process of building the top material. The following are two X-ray photoelectron spectroscopy (XPS) test experiments to prove and the concept of transition layer to understand the SEE phenomenon of multilayer materials.

**Fig.15:**
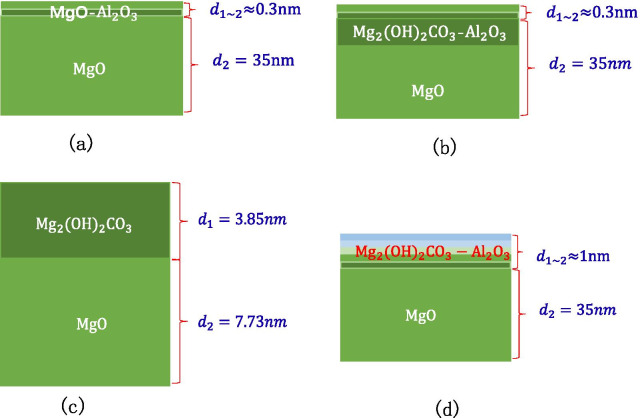
Schematic diagram of XPS test experiment sample, **a** shows the information of MgO/Al_2_O_3_ (on the silicon wafer, grow 35 nm-MgO, and then grow 0.3 nm-Al_2_O_3_), **b** shows the information of deliquescent MgO/Al_2_O_3_ (on the silicon wafer, grow 35 nm-MgO, and then grow 0.3 nm-Al_2_O_3_), **c** shows the information of deliquescent MgO (on the silicon wafer, grow 11 nm-MgO), **d** shows the information of deliquescent MgO/Al_2_O_3_ (on the silicon wafer, grow 35 nm-MgO, and then grow 1 nm-Al_2_O_3_)

**Fig. 16:**
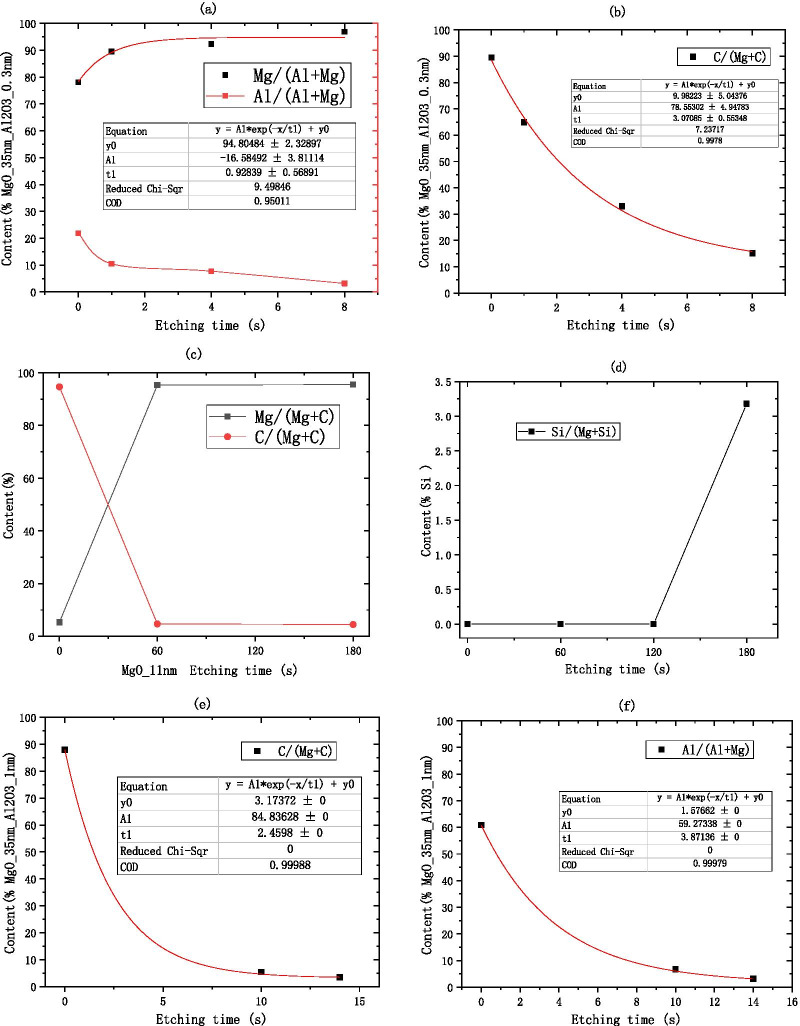
Atomic concentration percentage of C, Al, Si elements relative to Mg element obtained by XPS. **a** Shows the Al element information of deliquescent MgO/Al_2_O_3_ (on the silicon wafer, grow 35 nm-MgO, and then grow 0.3 nm-Al_2_O_3_), **a** shows the C element information of deliquescent MgO/Al_2_O_3_ (on the silicon wafer, grow 35 nm-MgO, and then grow 0.3 nm-Al_2_O_3_), **c** shows the C element information of deliquescent MgO (on the silicon wafer, grow 11 nm-MgO), **d** shows the Si element information of deliquescent MgO (on the silicon wafer, grow 11 nm-MgO), **e** shows the C element information of deliquescent MgO/Al_2_O_3_ (on the silicon wafer, grow 35 nm-MgO, and then grow 1 nm-Al_2_O_3_). **f** shows the Al element information of deliquescent MgO/Al_2_O_3_ (on the silicon wafer, grow 35 nm-MgO, and then grow 1 nm-Al_2_O_3_)

XPS test experiment 1:

First, the sample (0.3 nm ${\text{Al}}_{2} {\text{O}}_{3}$ grown on MgO) in the air for 1 year are tested for XPS as shown in Fig. 15a. We use an Ar ion gun to etch the surface of the material, and then test the various elements in the material by XPS. The two are alternately performed. The etching depth is controlled by controlling the etching time, and the relative atomic concentration percentage changes of various elements are obtained by XPS. Al element is almost undetectable after 8 s of etching as shown in Fig. 16a. The etching rate of ${\text{Al}}_{2} {\text{O}}_{3}$ is known, ${\text{Etching}}\,{\text{rate}}_{{{\text{Al}}_{2} {\text{O}}_{3} }} = 0.7{\text{{\AA}/s}}$,

$$\begin{aligned} & {\text{Etching}}\_{\text{Thickness}}_{{{\text{Al}}_{2} {\text{O}}_{3} }} = {\text{Etching rate }}_{{{\text{Al}}_{2} {\text{O}}_{3} }} *{\text{Etching time}}_{{{\text{Al}}_{2} {\text{O}}_{3} }} = 0.7\,{\text{{\AA}/s}}\,*\,8\,{\text{s}} = 5.6{\text{\AA}} \\ & {\text{Cycle}}\_{\text{Thickness}}_{{{\text{Al}}_{2} {\text{O}}_{3} }} = 1.29\,{\text{{\AA}/cycle}}\,*3\,{\text{cycle}} = 3.87{\text{\AA}} \\ & {\text{Etching}}\_{\text{Thickness}}_{{{\text{Al}}_{2} {\text{O}}_{3} }} > {\text{Cycle}}\_{\text{Thickness}}_{{{\text{Al}}_{2} {\text{O}}_{3} }} \\ \end{aligned}$$

Therefore, it shows that ${\text{Al}}_{2} {\text{O}}_{3}$ must exist in the MgO part, that is, ${\text{Al}}_{2} {\text{O}}_{3}$ destroys the lattice state of the MgO surface. ${\text{Al}}_{2} {\text{O}}_{3}$ forms a finite solid solution in MgO [30]. At this time, the experimentally measured SEE level increased. As we all know, the higher the SEE level, the better the insulation of the material. Due to the destruction of the surface lattice, the surface layer of MgO is more insulating, which further confirms the process of destroying the underlying material in the concept of the transition layer.

According to the results of the SEE experiment, the SEE level has dropped significantly. A small amount of ${\text{Al}}_{2} {\text{O}}_{3}$ in the top layer cannot protect the MgO in the bottom layer. MgO is still deliquescent in the air. The air contains ${\text{O}}_{2} ,{\text{H}}_{2} {\text{O}},{\text{CO}}_{2} ,{\text{CO}},{\text{N}}_{2}$, etc. When air enters MgO, the reaction of MgO and ${\text{CO}}_{2}$ and ${\text{H}}_{2} {\text{O}}$ proceeds at the same time.

$$\begin{aligned} & {\text{MgO}} + {\text{H}}_{2} {\text{O}} = {\text{Mg}}\left( {{\text{OH}}} \right)_{2} \\ & {\text{MgO}} + {\text{CO}}_{2} = {\text{MgCO}}_{3} \\ & {\text{Mg}}\left( {{\text{OH}}} \right)_{2} + {\text{CO}}_{2} \rightleftharpoons {\text{MgCO}}_{3} + {\text{H}}_{2} {\text{O}} \\ & 2{\text{MgO}} + 2{\text{H}}_{2} {\text{O}} + {\text{CO}}_{2} = {\text{Mg}}_{2} \left( {{\text{OH}}} \right)_{2} {\text{CO}}_{3} \\ \end{aligned}$$

The above four chemical reactions occur, the deliquescent reaction of air and MgO is mainly the reaction of MgO and ${\text{CO}}_{2}$ and ${\text{H}}_{2} {\text{O}}$ to produce ${\text{MgCO}}_{3}$ and ${\text{Mg}}_{2} \left( {{\text{OH}}} \right)_{2} {\text{CO}}_{3}$. As long as the prepared MgO is exposed to the air, ${\text{Mg}}\left( {{\text{OH}}} \right)_{2}$ will be produced. After being placed in the air for 28 days, ${\text{MgCO}}_{3}$ is the main product [31]. Because the tested MgO sample needs to be transferred to the SEE test equipment, the actual test is the SEE level of MgO–${\text{Mg}}\left( {{\text{OH}}} \right)_{2}$. Main reason for the decrease in SEE level is the ${\text{Mg}}_{2} \left( {{\text{OH}}} \right)_{2} {\text{CO}}_{3}$ and MgCO_3_ produced by deliquescent. Therefore, when using XPS, C can be selected as the calibration element for the deliquescent depth of MgO in the air. As shown in Fig. 16b, after 8 s of etching, no Al content is detected, but C content is still detected, indicating that the MgO in the bottom layer continues to deliquesce and is not protected by a small amount of ${\text{Al}}_{2} {\text{O}}_{3}$ as shown in Fig. 15b.

XPS test experiment 2:

First, the MgO sample in the air for 1 year are tested for XPS. After 1 min of etching, there was almost no C element as shown in Fig. 16c, indicating that the thickness of the dense ${\text{Mg}}_{2} \left( {{\text{OH}}} \right)_{2} {\text{CO}}_{3}$ film formed was the thickness of 1 min of etching.

After etching for 3 min, the sample begins to show Si element as shown in Fig. 16d, the etching rate of MgO and the thickness of ${\text{Mg}}_{2} \left( {{\text{OH}}} \right)_{2} {\text{CO}}_{3}$ film can be calculated through these data.

$$\begin{aligned} & {\text{Etching rate }}_{{{\text{MgO}}}} = \frac{{{\text{Thickness}}_{{{\text{MgO}}}} }}{{{\text{Etching time}}_{{{\text{MgO}}}} }} = \frac{{11.58\,{\text{nm}}}}{{180\,{\text{s}}}} = 0.643{\text{{\AA}/s}} \\ & {\text{Etching}}\_{\text{Thickness}}_{{{\text{Mg}}_{2} \left( {{\text{OH}}} \right)_{2} {\text{CO}}_{3} }} \approx {\text{Etching}}\_{\text{Thickness}}_{{{\text{MgO}}}} \\ & \quad = {\text{Etching rate }}_{{{\text{MgO}}}} \,*\,{\text{Etching time}}_{{{\text{MgO}}}} = 0.643{\text{\AA}}/{\text{s*}}60\,{\text{s}} \approx 3.85\,{\text{nm}} \\ \end{aligned}$$

The 3.85 nm ${\text{Mg}}_{2} \left( {{\text{OH}}} \right)_{2} {\text{CO}}_{3}$ film layer acts as an air barrier layer to prevent further deliquescent of deep MgO as shown in Fig. 15c.

When 1 nm ${\text{Al}}_{2} {\text{O}}_{3}$ is grown on MgO, the XPS test data show that there is basically no C content and no Al content in the sample after the etching time of 14 s as shown in Fig. 16e, f.

$${\text{Etching}}\_{\text{Thickness}}_{{{\text{Al}}_{2} {\text{O}}_{3} }} = {\text{Etching rate }}_{{{\text{Al}}_{2} {\text{O}}_{3} }}* {\text{Etching time}}_{{{\text{Al}}_{2} {\text{O}}_{3} }} = 0.7\,{\text{{\AA}/s}}\,*\,14\,{\text{s}} = 9.8{\text{\AA}}$$

It can be known by testing the C content that the depth of air penetration into the material is about 1 nm at this time. According to the concept of the transition layer, there are two kinds of materials at the interface to form the process of destroying the bottom layer material and constructing the top layer material. At the interface, ${\text{Al}}_{2} {\text{O}}_{3}$ destroys the crystal lattice on the surface of MgO. In order to prevent excessive infiltration of air, a complete ${\text{Al}}_{2} {\text{O}}_{3}$ atomic level is formed at least at 1 nm. When a complete ${\text{Al}}_{2} {\text{O}}_{3}$ atomic layer is not formed, the infiltration of air into the material cannot be prevented as in Example 1 above. The ${\text{Al}}_{2} {\text{O}}_{3}$ and ${\text{ Mg}}_{2} \left( {{\text{OH}}} \right)_{2} {\text{CO}}_{3}$ in the inner layer are mixed to help MgO form a dense air barrier layer in advance as shown in Fig. 15d.

**Fig. 17:**
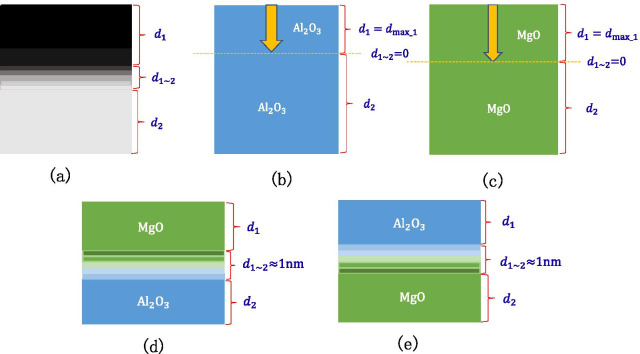
**a** Schematic diagram of the transition layer of the double layer structure, **b** schematic diagram of the Al_2_O_3_ transition layer and incident electron depth, **c** schematic diagram of the MgO transition layer and incident electron depth, **d** schematic diagram of the Al_2_O_3_/MgO transition layer, **e** schematic diagram of the MgO/Al_2_O_3_ transition layer

The concept of transition layer understands the SEE phenomenon of multilayer materials:

The schematic diagram shown in Fig. 17a shows the concept of the transition layer, The thickness of the top layer material is $d_{1}$, the thickness of the bottom layer material is $d_{2}$ and the thickness of the transition layer is $d_{1\sim 2}$.The schematic diagram is shown in Fig. 17b, c when there is enough thick ${\text{Al}}_{2} {\text{O}}_{3}$ or MgO, the incident electron depth is $d_{{{\text{max}}\_1}}$, and there is no transition layer between ${\text{Al}}_{2} {\text{O}}_{3}$ and ${\text{Al}}_{2} {\text{O}}_{3}$ (there is no transition layer between MgO and MgO), that is, the thickness of the transition layer is 0. Through XPS test experiment 2, we get that the thickness of the transition layer between MgO and ${\text{Al}}_{2} {\text{O}}_{3}$ is 1 nm as shown in Fig. 17d, e.

When the top layer material in the double-layer structure is MgO, the thickness of the MgO that reaches the saturated SEE level is different when the bottom layer material is different. If electrons are incident on the bottom layer material, the SEE level of the bottom layer material is low and cannot reach the saturated SEE level. Therefore, to reach the saturation SEE level, a complete MgO incident electron path needs to be formed. When the bottom layer material is different, such as Si or ${\text{Al}}_{2} {\text{O}}_{3}$, the thickness of the transition layer will be different, so the top layer MgO shows a different thickness.

**Fig. 18:**
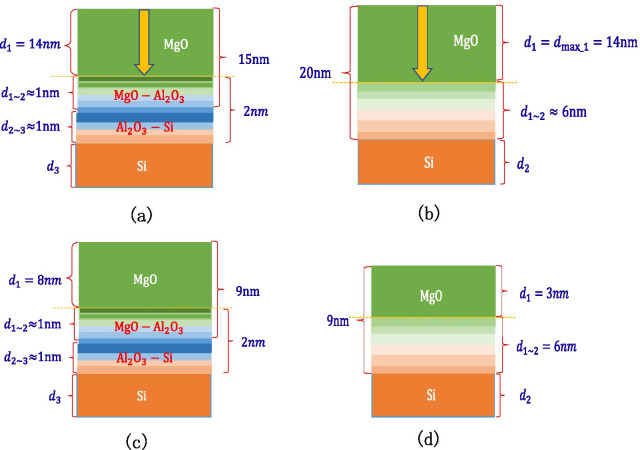
Schematic diagram of the thickness of each layer of a multilayer structure, **a** shows the thickness of Al_2_O_3_/MgO (on the silicon wafer, grow 2 nm-Al_2_O_3_, and then grow 15 nm-MgO), **b** shows the thickness of MgO (on the silicon wafer, grow 20 nm-MgO), **c** shows the thickness of Al_2_O_3_/MgO (on the silicon wafer, grow 2 nm-Al_2_O_3_, and then grow 9 nm-MgO), **d** shows the thickness of MgO (on the silicon wafer, grow 9 nm-MgO)

It is found through experiments that a sample that grows 2 nm ${\text{Al}}_{2} {\text{O}}_{3}$ on a Si wafer and then grows 15 nm MgO can reach the SEE level of MgO saturation. Knowing that the thickness of the MgO–${\text{Al}}_{2} {\text{O}}_{3}$ transition layer is 1 nm, it can be inferred that the thickness of the ${\text{Al}}_{2} {\text{O}}_{3}$–Si transition layer is 1 nm, and the maximum depth of incident electrons of MgO is 14 nm as shown in Fig. 18a. It is found through experiments that the sample of 20 nm MgO grown on the Si wafer can reach the SEE level of MgO saturation. It has been inferred that the maximum depth of incident electrons of MgO is 14 nm, so the thickness of the MgO–Si transition layer can be calculated to be 6 nm as shown in Fig. 18b. Therefore, it can be explained that the SEE level of growing 2 nm ${\text{Al}}_{2} {\text{O}}_{3}$ on Si wafer and then growing 9 nm MgO is higher than the SEE level of 9 nm MgO growing on Si wafer. This is because the thickness of the MgO–${\text{Al}}_{2} {\text{O}}_{3}$ transition layer is thinner than that of the MgO–Si transition layer. The actual MgO thickness of 8 nm involved in incident electrons is much thicker than 3 nm as shown in Fig. 18c, d.

**Fig. 19:**
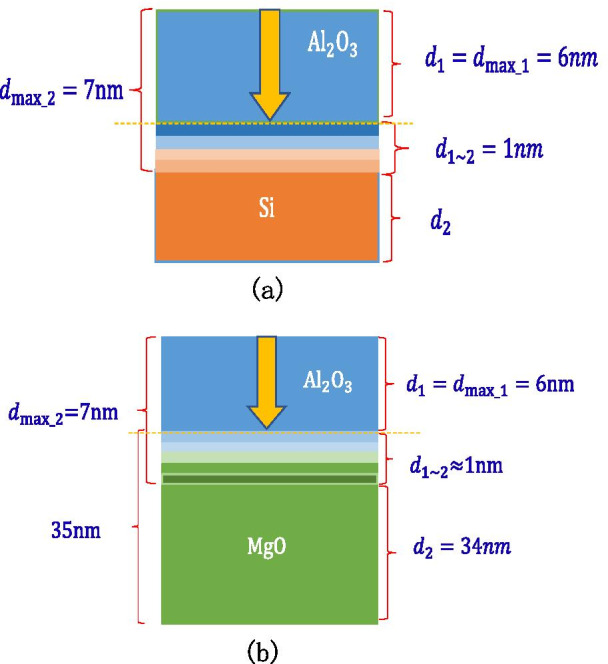
Schematic diagram of the thickness of each layer of a multilayer structure, **a** shows the thickness of Al_2_O_3_ (on the silicon wafer, grow 7 nm-Al_2_O_3_), **b** shows the thickness of MgO/Al_2_O_3_ (on the silicon wafer, grow 20 nm-MgO, and then grow 7 nm-Al_2_O_3_)

It can be seen through experiments that growing 7 nm ${\text{Al}}_{2} {\text{O}}_{3}$ on Si wafers can reach the SEE level of ${\text{Al}}_{2} {\text{O}}_{3}$ saturation, so it can be calculated that the maximum depth of incident electrons of ${\text{Al}}_{2} {\text{O}}_{3}$ is 6 nm; growing 7 nm ${\text{Al}}_{2} {\text{O}}_{3}$ on 35 nm MgO can reach the SEE level of ${\text{Al}}_{2} {\text{O}}_{3}$ saturation, the thickness of the MgO-${\text{Al}}_{2} {\text{O}}_{3}$ transition layer is 1 nm, and the maximum depth of incident electrons of ${\text{Al}}_{2} {\text{O}}_{3}$ is calculated again to be confirmed by 6 nm, as shown in Fig. 19a, b.

## Conclusions

In conclusion, we designed a global-shaped structure device for testing the SEE of the material and propose to use low-energy secondary electrons instead of low-energy electron beam for neutralization to measure the insulating material. We designed the emission layer of the electron multiplier with the idea of building a house to study the relationship between ${\text{Al}}_{2} {\text{O}}_{3}$ and MgO. We propose the nearest neighbor SEE ratio and use this to divide the SEE incident electron energy of the material into the high-energy region, the middle-energy region and the low-energy region. We have obtained four empirical formulas for SEE and thickness by studying ${\text{Al}}_{2} {\text{O}}_{3}$, MgO, MgO/${\text{Al}}_{2} {\text{O}}_{3}$,${\text{ Al}}_{2} {\text{O}}_{3}$/MgO. We propose to use the concept of transition layer for SEE interpretation of multilayer materials and obtained the optimal ${\text{Al}}_{2} {\text{O}}_{3}$/MgO/${\text{Al}}_{2} {\text{O}}_{3}$ three-layer structure thickness suitable for electron multiplier through formula analysis and experimental experience. The thin film with this structure can maintain a high SEE level for a long time. This new emission layer will have broad application prospects in the channel electron multiplier (CEM), microchannel plate (MCP), independent electron multiplier and other devices.

## Data Availability

The authors do not wish to share their data. Because the authors have academic competition with other institutions. The authors want to protect their academic achievements and seek research funding for future research.
